# Pseudotumor cerebri syndrome in children with systemic lupus erythematosus: case series and review

**DOI:** 10.1186/s12969-022-00688-5

**Published:** 2022-04-15

**Authors:** Taha Moussa, Moussa Abdelhak, Cuoghi Edens

**Affiliations:** 1 Pediatrics, Boston Children’s Hospital, Boston, USA; 2 Mill Road Surgery, Cambridge, UK; 3 Pediatric Rheumatology, Pediatrics, University of Chicago Medicine, Chicago, USA

**Keywords:** Systemic lupus erythematosus, Pseudotumor cerebri, Intracranial hypertension, Headache

## Abstract

**Background:**

Systemic lupus erythematosus (SLE) is a chronic autoimmune inflammatory disease that typically affects multiple organs and can lead to potentially fatal complications. Central nervous system (CNS) involvement in SLE is common, especially in children, and can present nonspecifically with various neuropsychiatric manifestations, described as neuropsychiatric SLE (NPSLE). Chronic headache is a common feature of NPSLE, secondary to increased intracranial pressure (also called pseudotumor cerebri (PTC)) due to inflammation or medication. Here, we highlight the importance of evaluating refractory headache (HA) in SLE patients to rule out PTC as a cause of severe morbidity.

**Methods:**

Single tertiary care pediatric center case series of 8 children who developed NPSLE in the form of intracranial hypertension at or after SLE diagnosis.

**Conclusion:**

Neurologic and ophthalmologic evaluation of refractory HA in patients with SLE, especially children, is warranted to decrease the burden of the disease and rule out treatable causes like PTC.

## Background

Systemic lupus erythematosus (SLE) is a chronic autoimmune inflammatory disease that typically affects multiple organ systems and can lead to potentially fatal complications. Management of moderate to severe SLE is often challenging and requires chronic use of immunotherapy and systemic corticosteroids (CS). Central nervous system (CNS) involvement in SLE is common, especially in children, and can present nonspecifically with various neuropsychiatric manifestations; collectively termed neuropsychiatric SLE (NPSLE) [1, 2]. Pediatric rheumatologists have adopted the American College of Rheumatology (ACR) adult guidelines for NPSLE nomenclature; however, NPSLE is the least understood feature of lupus disease across age groups. Headache (HA), cognitive dysfunction, and cerebrovascular disease are the most common manifestations of NPSLE in adults and children [1–4] but are not part of the current ACR classification criteria.

Pseudotumor cerebri (PTC) is a term used to describe intracranial hypertension, which can be idiopathic or secondary to underlying etiology such as abnormal brain parenchyma, venous thrombosis, space-occupying lesion, medication, infection, inflammation, and other related medical conditions. It was described in 1937 as "benign" intracranial hypertension despite reported vision loss [5]. Acute episodes usually present with refractory HA, sixth nerve palsy, and/or papilledema. Obese adolescent females are most commonly affected, and more frequent relapses are reported in the prepubertal age [6]. In 2013, Friedman et al. revised the diagnostic criteria for PTC syndrome (PTCS) according to their body habitus and sedation. In a correctly performed lumbar puncture (LP), they defined the threshold of cerebrospinal fluid (CSF) opening pressure in children versus adults according to their body habitus and sedation in a properly performed lumbar puncture (LP). According to their definition, ruling out underlying possible secondary etiologies through normal neuroimaging, neurologic examination, and CSF composition to rule out secondary etiologies is essential for PTCS diagnosis [7].

Cases of PTCS in children with SLE have been reported; however, there is no evidence to prove an association between both [8–12]. Here, we describe a case series of eight children who developed NPSLE in the form of PTCS after diagnosis or as the first manifestation of SLE to highlight the importance of proper neurologic and ophthalmologic evaluation of refractory HA in children with SLE.

## Methods

This is a single pediatric tertiary center (University of Chicago Medicine) case series of children who developed PTCS at or after their SLE diagnosis. Electronic medical records for patients < 18 years old who had an ICD-9 or ICD-10 codes for SLE and PTCS from 2010 to 2021 were identified reviewed by the research team. Among 240 pediatric SLE patients cared for at that time, 8 were diagnosed with PTCS at or after being diagnosed with SLE by a pediatric rheumatologist. Demographics and clinical data were reviewed at both SLE and PTCS diagnoses.

## Results

The majority of the patients in this case series were adolescent females of either African American (AA) (5/8) or Hispanic (2/8) ethnicity (Table 1). Refractory HA was exhibited in 100% of patients, 5/8 with prolonged HA course (≥ 9 months) and multiple inpatient admissions. Obesity was a common finding, with an obese body max index found in 5/8 at PCTS diagnosis. SLE Disease Activity Index (SLEDAI) is used to connote the severity; a score of ≥ 3 is considered mild/moderate, and a score ≥ 12 points are severe [13]. 8/8 (100%) of patients had severe SLE disease activity at PTCS diagnosis. Regarding serologic findings, 4/8 patients had positive antiphospholipid antibodies, 7/8 patients had low complement levels, and 6/8 patients had elevated inflammation markers. 7/8 patients had lupus nephritis, with 6/8 having class V lupus nephritis. While renal function was normal in all patients, 3/8 patients had nephrotic range proteinuria and low serum albumin level. Abnormal neurologic examination and neuroimaging were reported in 4/8 patients; however, only 1/8 patients had abnormal CSF composition. Although the first CSF opening pressure was borderline in 3/8 patients, the diagnosis was explicitly supported in those patients by failing other measures to alleviate their refractory HA, including controlling the underlying active lupus disease and remarkable improvement after therapeutic LP and acetazolamide treatment. Papilledema was absent in 3/8 patients on examination of the optic nerve. Acetazolamide was the primary treatment; it was successfully discontinued in 2 patients with no subsequent HA episodes.

**Table 1:**
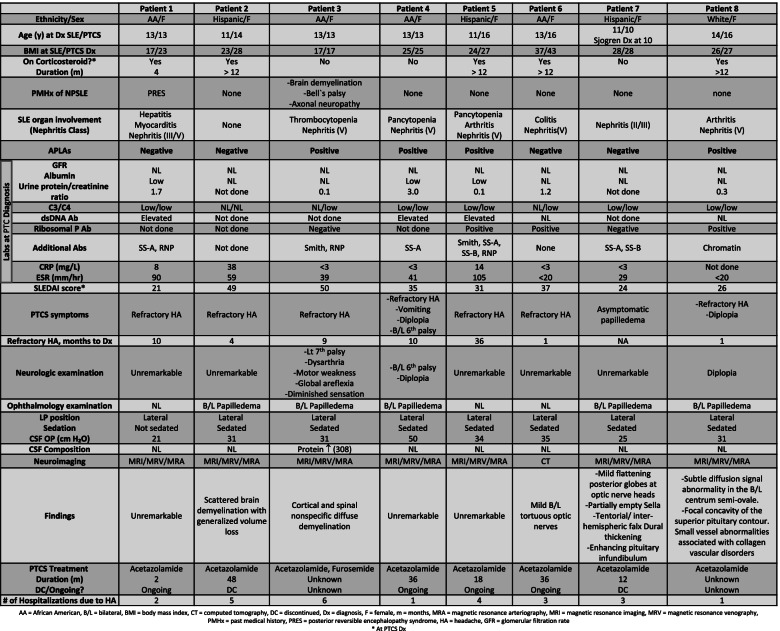
Pediatric Systemic Lupus Erythematosus with Pseudotumor Cerebri Subject Characteristics, Evaluation, Treatment and Outcomes

## Discussion

NPSLE continues to be a descriptive term for CNS involvement in new or established SLE disease without clear diagnostic criteria [9]. The prevalence of CNS involvement in pediatric SLE is estimated as a wide range between ~ 20–95% [1, 2, 14, 15]. It was described by several clinical features and correlated with other immune-mediated CNS diseases, shown in Table 2. Several reports proposed a possible correlation between serum and CSF autoantibodies and NPSLE; however, they lacked adequate sensitivity and specificity [16] and were not performed in our case series. In children, HA is the most common manifestation of NPSLE, followed by mood disorders and cognitive dysfunction [1]. A retrospective study of children showed that NPSLE commonly starts early in the disease course and is not necessarily associated with the overall disease activity of SLE [14]. Thus, approaching refractory HA in SLE is challenging due to the high prevalence of HA in the general population and multifactorial etiologies of systemic inflammation, corticosteroid side effects, and CNS disease.

**Table 2:**
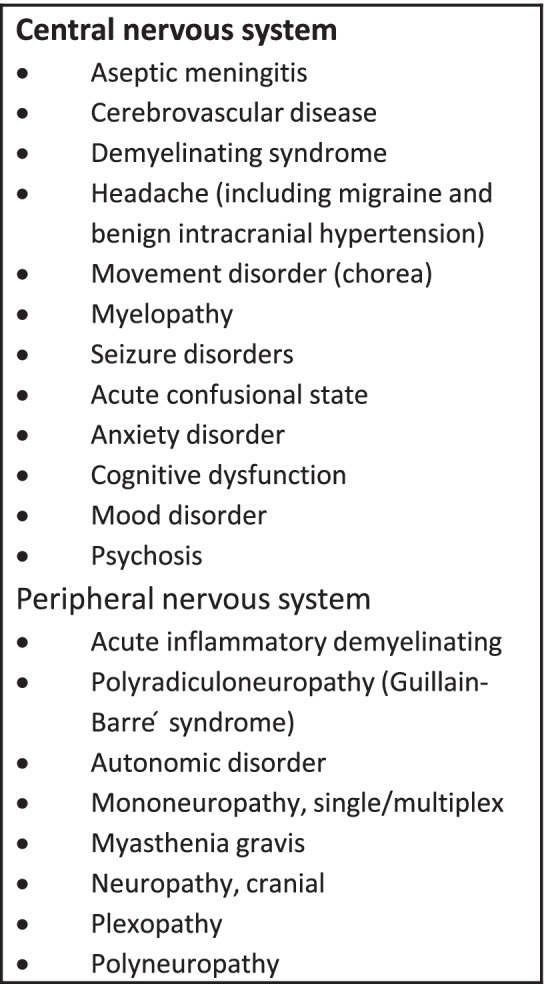
Neuropsychiatric Syndromes Observed in SLE [1]

Benign intracranial hypertension is included in the list of NPSLE nomenclature as a cause of severe, disabling HA not responsive to narcotics [3]. In addition to HA, papilledema and/or 6^th^ nerve palsy are common manifestations of PTCS without evidence of underlying etiology [6]. Visual loss is the most feared complication of PTCS if not treated early. Vision loss was reported in two asymptomatic prepubertal girls due to PTCS after growth hormone treatment [17]. Due to the gradual increase of intracranial pressure (ICP), PTCS patients can be asymptomatic or without papilledema, as shown in our case series. While not present in all PTCS patients in our study, fundus examination of the eye at regular follow-up visits for SLE patients may be a convenient tool to detect papilledema in asymptomatic patients. However, proper neurological examination and a team approach are warranted for any SLE patient with refractory HA or other neurologic manifestations, and further workup is to be considered accordingly.

In an adequately performed LP, CSF opening pressure is still the gold standard for intracranial hypertension diagnosis. The last revision of PTCS diagnostic criteria in 2013 described the PTCS nomenclature to include both primary and secondary intracranial hypertension and considered both definite and probable PTCS. The criteria highlighted the threshold for CSF opening pressure according to the body habitus and use of sedation; 28 cm H_2_O for obese and/or sedated children who have either papilledema and/or sixth nerve palsy. A lower CSF opening pressure threshold (25 cm H_2_O) is used only for not sedated or obese children. In addition, exclusion of any underlying etiology through normal neuroimaging, neurologic examination, and CSF composition should be pursued [7]. NPSLE patients may not fit these criteria since they are at higher risk of vasculitis and brain parenchyma abnormalities, abnormal neuroimaging, neurologic examination, and CSF composition. According to the criteria, 2/8 of our series had normal CSF opening pressure; however, they failed other measures, including controlling their active lupus disease and HA treatment. They improved remarkably after therapeutic LP and acetazolamide therapy. CSF analysis is to be sent to rule out inflammation and infection since SLE patients are often chronically immunosuppressed, and systemic inflammatory and infectious symptoms may not be obvious until a late stage. CNS demyelination, present in 1 patient with normal and 1 patient with high CSF opening pressure, has been described in NPSLE and should not delay the evaluation of PTCS.

In 1999, a consensus statement by the American College of Rheumatology [3], "benign intracranial hypertension" was listed as a manifestation of lupus affecting the central nervous system and resulting in severe, disabling HA not responsive to narcotics. As it was later recognized that this condition is not always "benign", the preferred terminology for the syndrome of increased intracranial pressure in lupus has changed over time to "pseudotumor cerebri" or "pseudotumor cerebri syndrome." Other manifestations of PTCS include papilledema, cranial nerve palsies, olfactory dysfunction, tinnitus, and cognitive defects [6]. Patients with PTCS may be asymptomatic [17], presumably due to the gradual increase in intracranial pressure, and may not even have papilledema [3], as demonstrated in our series. Nevertheless, a fundoscopic examination is essential for routine follow-up visits for symptomatic and asymptomatic SLE patients. If abnormalities are suspected, the patient should be urgently evaluated by an ophthalmologist with expertise in neurologic disorders.

A more comprehensive neurologic exam and a team approach are necessary to evaluate lupus patients with refractory HA or other neurologic manifestations. Since both innate immune defects and treatment with immunosuppressive medications increase the risk for infection in lupus patients, neuroimaging (either CT scan or MRI with vessel imaging) is routinely performed to assess for mass lesions, thrombosis, and diffuse CNS inflammation as causes of HA. Likewise, following a correctly performed LP, CSF analysis is essential to detect abnormally high intracranial pressure and identify possible infection. Because many lupus patients are chronically immunosuppressed, systemic symptoms of CNS infection may be subtle.

Due to several patient and disease-specific factors, children with SLE are at increased risk of developing intracranial hypertension. Obesity is a common side effect of corticosteroids, one of the mainstay treatments of SLE induction and disease flare, and both are independent risk factors for PTCS [18]. In addition, both SLE and PTCS are more common in adolescent females than in males and other female age groups [19, 20]. Furthermore, SLE patients are at higher risk for coagulopathy than normal children secondary to SLE disease activity, antiphospholipid antibodies, and nephritis [21]. Coagulopathy can lead to thrombotic and ischemic cerebrovascular disease and inflammation of brain parenchyma, a recognized presentation of NPSLE and a leading cause of secondary intracranial hypertension [22]. In our series, 100% had severe SLE disease activity as determined by SLEDAI score, and 50% had positive APLAs at PTCS diagnosis. Furthermore, the majority (87.5%) had lupus nephritis, 75% had class V lupus nephritis, and 37.5% had nephrotic range proteinuria with low serum albumin levels.

According to the most recent revision of PTCS diagnostic criteria in 2013 [7], thresholds for abnormal CSF opening pressure vary according to body habitus and procedural use of sedation as follows: > 28 cm H_2_O for obese and/or sedated children, and > 25 cm H_2_O for children who are not sedated or obese. Many other factors can affect opening pressure; for example, corticosteroids, obesity, position, and sedation during the procedure, so a borderline opening pressure does not exclude PTC. 2/8 patients in our series had borderline CSF opening pressure according to the criteria; nonetheless, one of these patients had an abnormal MRI. Both had HA refractory to SLE and HA management. Both subjects experienced improvements in HA following therapeutic lumbar puncture and acetazolamide treatment.

In our case report, intracranial hypertension was not recognized as a cause of refractory HA in children with SLE and led to repeat emergency room visits and hospitalizations. Of note, prior to their diagnostic admission, these health care interactions were more often at hospitals without pediatric subspecialists. In our patients, therapeutic LP and acetazolamide were successful and convenient measures to control their ICP and prevent further progression. Vision loss has not been reported by any subject in our case series.

## Conclusion

Intracranial hypertension is underreported and likely under-recognized in children with SLE. Regardless of the term used to describe intracranial hypertension in SLE (primary, secondary, NPSLE, PTC, or PTCS), it is still a treatable cause of significant morbidity among lupus patients. Vision loss is the most feared complication of intracranial hypertension, which can develop even in asymptomatic patients. A high index of suspicion should be maintained for PTCS in an SLE patient with a HA. Proper evaluation of intracranial hypertension through complete neurologic examination, ophthalmological examination, neuroimaging, and LP with opening pressure should be pursued in any SLE patient with a refractory HA or sixth nerve palsy. Lupus nephritis, active SLE, and corticosteroid use may play a role in the incidence of PTCS in pediatric SLE patients. Regular fundus examination during follow-up visits is a convenient tool to detect asymptomatic intracranial hypertension in SLE patients. In addition to control of underlying lupus disease, acetazolamide and therapeutic lumbar puncture are successful measures to treat high ICP in NPSLE. CNS involvement is common in children with SLE, and PTC can be the first presentation.

## Data Availability

The data used during the current study are available from the corresponding author upon reasonable request.

## Abbreviations

SLE: Systemic lupus erythematosus
NPSLE: Neuropsychiatric lupus
PTC: Pseudotumor cerebri
ICH: Intracranial hypertension
PTCS: Pseudotumor cerebri syndrome
ACR: American college of rheumatology
LP: Lumbar puncture
SLEDAI: SLE disease activity index
GFR: Glomerular filtration rate
